# A Randomized Control Trial Evaluating an Online Mindful Parenting Training for Mothers With Elevated Parental Stress

**DOI:** 10.3389/fpsyg.2019.01550

**Published:** 2019-07-17

**Authors:** Eva S. Potharst, Myrthe G. B. M. Boekhorst, Ivon Cuijlits, Kiki E. M. van Broekhoven, Anne Jacobs, Viola Spek, Ivan Nyklíček, Susan M. Bögels, Victor J. M. Pop

**Affiliations:** ^1^UvA Minds, Academic Outpatient (Child and Adolescent) Treatment Center, University of Amsterdam, Amsterdam, Netherlands; ^2^Research Institute of Child Development and Education, University of Amsterdam, Amsterdam, Netherlands; ^3^Department of Medical and Clinical Psychology, Tilburg University, Tilburg, Netherlands; ^4^Department of Obstetrics and Gynaecology, Máxima Medical Centre, Veldhoven, Netherlands; ^5^Department of Psychiatry, Erasmus MC, University Medical Centre, Rotterdam, Netherlands; ^6^Department of Psychology, Fontys University of Applied Sciences, Eindhoven, Netherlands; ^7^Developmental Psychology, University of Amsterdam, Amsterdam, Netherlands

**Keywords:** mindful parenting, online intervention, parental stress, early intervention, behavior problems

## Abstract

**Objectives:**

The prevalence of maternal stress in early years of parenting can negatively impact child development. Therefore, there is a need for an early intervention that is easily accessible and low in costs. The current study examined the effectiveness of an 8-session online mindful parenting training for mothers with elevated levels of parental stress.

**Methods:**

A total of 76 mothers were randomized into an intervention (*n* = 43) or a waitlist control group (*n* = 33). The intervention group completed pretest assessment prior to the online intervention. Participants completed a post intervention assessment after the 10 weeks intervention and a follow-up assessment 10 weeks later. The waitlist group completed waitlist assessment, followed by a 10-week waitlist period. After these 10 weeks, a pretest assessment took place, after which the waitlist group participants also started the intervention, followed by the posttest assessment. Participating mothers completed questionnaires on parental stress (parent-child interaction problems, parenting problems, parental role restriction) and other maternal (over-reactive parenting discipline, self-compassion, symptoms of depression and anxiety) and child outcomes (aggressive behavior and emotional reactivity) while the non-participating parents (father or another mother) were asked to also report on child outcomes.

**Results:**

The online mindful parenting intervention was shown to be significantly more effective at a 95% level than a waitlist period with regard to over-reactive parenting discipline and symptoms of depression and anxiety (small and medium effect sizes), and significantly more effective at a 90% level with regard to self-compassion, and mother-rated child aggressive behavior and child emotional reactivity (small effect sizes). The primary outcome, parental stress, was found to have a 95% significant within-group effect only for the subscale parental role restriction (delayed small effect size improvement at follow-up). No significant improvements on child outcomes were found for the non-participating parent.

**Conclusion:**

To conclude, the results provide first evidence that an online mindful parenting training may be an easily accessible and valuable intervention for mothers with elevated levels of parental stress.

## Introduction

A child’s social, emotional, behavioral, cognitive and physical development in the first years of life is an important foundation for later development (Briggs-Gowan et al., 2006; Feldman and Eidelman, 2009; Bornstein et al., 2010). The development of children is supported in a positive manner when they have the possibility to build a secure relationship with their parents, in which the parents are supportive and sensitive (Deater-Deckard, 2005; Bernier et al., 2010). An important risk factor for problems in parenting behavior and the parent-child relationship is parental stress (Mills-Koonce et al., 2011; McMahon and Meins, 2012). Parental stress does not only have negative consequences for child development via certain parenting practices and behaviors, but is also directly related to problems in social, emotional and behavioral development (Anthony et al., 2005; Crnic et al., 2005). Even when parents do not fall into certain “parenting traps” associated with elevated levels of parental stress, children may be affected by parental stress through the emotional climate in the family or through observational learning of parental emotion regulation (Morris et al., 2007).

Parental stress is defined as the aversive psychological reaction to the demands of parenthood (Deater-Deckard, 1998). Child, parent, family and environmental factors influence the level of stress that parents experience (Östberg and Hagekull, 2000). A prospective study that investigated parental stress and child behavior problems in families with children aged 3 to 9, showed that a high level of child behavior problems is a risk factor for parental stress and vice versa (Anthony et al., 2005; Neece et al., 2012). A vicious cycle with increasing levels of both parental stress and child behavior problems can lead to negative consequences for the quality of the parent-child interaction and the security of their relationship (Ciciolla et al., 2014; Lewallen and Neece, 2015).

Parents differ in their capacity to deal with and regulate parental stress (Leerkes et al., 2017). Parents with high levels of stress and low regulatory capacity, have a higher risk of being “over-reactive” toward their children in difficult parenting situations (Prinzie et al., 2007; Lorber, 2012). Over-reactive parenting can be described as a parent’s tendency to respond with impatience and anger to problematic behavior of their children (Prinzie et al., 2007). Over-reactivity in parenting is found to be predictive of child externalizing problems (O’Leary et al., 1999; Miller-Lewis et al., 2006). A longitudinal study of families with adopted children (ages 9 to 27 months) showed that genetic risk for negative emotionality predicted child negative emotionality only when the adoptive mothers showed a high level of over-reactive parenting (Lipscomb et al., 2012).

Parents with mental health problems seem to be more susceptible to higher levels of parental stress. In the postpartum period, elevated symptoms of depression or anxiety were associated with elevated levels of parental stress (Crugnola et al., 2016). Mothers with postpartum depression continued to show elevated levels of parental stress when their child was 3 years of age (Milgrom et al., 2006). Not only parental stress, but also parental mental health problems have negative consequences for the parent-child relationship (Siegel and Hartzell, 2003). Therefore, a combination of mental health problems and parental stress may increase parents’ vulnerability in their parenting role. Although one could expect treatment of mental health problems to be beneficial not only for the mother’s well-being, but also with regard to parenting, the parent-child relationship, and the child’s well-being and development, this may not necessarily be the case (Milgrom et al., 2006; Kersten-Alvarez et al., 2011; Murray et al., 2014). Treatment of parents with a combination of mental health and parenting problems should not only focus on reducing their mental health problems, but also on reducing levels of parental stress, improving parental bonding to the child, and improving the quality of parent-child interaction.

Furthermore, parents with low levels of self-compassion have an increased likelihood of experiencing high levels of parental stress. An association between low self-compassion and parental stress was shown both in a community sample (Gouveia et al., 2016), and in parents of children with autism (Beer et al., 2013; Neff and Faso, 2015). The support that parents with a high level of self-compassion are able to obtain, may make them more resilient against parental stress (Neff and Faso, 2015), similar to the effect of social support on parental resilience (Horton and Wallander, 2001).

It has become clear how parental vulnerabilities (high psychopathology, low regulatory capacity and self-compassion), child vulnerabilities (difficult temperament, behavior problems), family and environmental factors contribute to parental stress. Specific developmental challenges associated with the age of the child may also play a role. Parents of toddlers are faced with the challenge of navigating between respecting the high need for autonomy in toddlers, and the high need for regulatory support (Early Child Care Research Network. [NICHD], 2004). Toddlers’ limited ability to regulate emotions and behavior may result in non-compliance, aggression, impulsivity and hyperactive behavior, which makes a certain level of parental stress normal in the toddler period. However, Deater-Deckard (1998) emphasized that even though some parental stress is normal, variation in both the normal and the extreme ranges of parental stress have been linked to adjustment in parents and children. Parental stress early in the child’s life has also been shown to be predictive of parental stress later in middle childhood (Östberg et al., 2007).

It is therefore important to provide parents who are experiencing elevated levels of parental stress with an intervention focused on coping with and reducing parental stress. In most parent training programs, a reduction of parental stress is achieved by teaching parents certain (cognitive behavioral) parenting techniques (Lundahl et al., 2006). However, Neece et al. (2012) posited the possibility of reducing parental stress by providing parents stress management trainings. Mindfulness training in the form of a mindfulness-based stress reduction training (MBSR; Kabat-Zinn, 1990) is being used world-wide for different kinds of stress-related complaints. Mindful parenting training is an adapted intervention that is specifically aimed at helping parents cope with, and regulate, their parental stress (Bögels et al., 2014; Potharst et al., in press a).

Mindful parenting can be defined as the ongoing process of intentionally bringing moment-to-moment, non-judgmental awareness as best one can to the unfolding of one’s own lived experience, including parenting (Kabat-Zinn and Kabat-Zinn, 1997). This non-judgmental moment-to-moment awareness can support parents in becoming aware of increasing levels of stress, accepting the situation and their own feelings, regulating their feelings, and making a more conscious decision instead of giving an impulsive reaction that is driven by stress. It can also aid parents in becoming more attentive toward their children, to what they communicate (both in words and by the non-verbal signals provoked), and in terms of emotional availability. Although Mindful Parenting training has mainly been applied to parents of children with psychopathology or developmental problems (Singh et al., 2007; Bögels et al., 2014; Meppelink et al., 2016; Emerson et al., in press), it has also been shown to be effective in reducing parental stress in a preventive setting (Potharst et al., in press a). A study by Potharst et al. (in press b) showed that a Mindful Parenting training adjusted for mothers with a toddler, the “Mindful with your toddler” training, was effective in reducing parental stress, as well as in improving mother-child interactions, and child behavior problems.

The fact that so many parents experience, or are at risk for high levels of parental stress when their child is at the toddler age calls for early interventions that are both easily accessible and low in costs. The use of internet has facilitated offering available interventions to large populations while keeping the societal costs low. Mindfulness-based interventions have also been adjusted to an internet format. This has additional benefits, such as reduced waiting time before the start of an intervention, freedom to pursue the intervention from home in one’s own time and pace, and anonymity (Spijkerman et al., 2016). In a meta-analysis, Spijkerman et al. (2016) showed that mindfulness-based internet interventions were not only effective in reducing stress (medium effect size), but also symptoms of depression and anxiety, as well as improving well-being and mindfulness (small effect sizes). In this meta-analysis, no mindfulness interventions for parents were included. Another meta-analysis on online non-mindfulness based parenting programs showed that online parenting interventions were effective in improving both parental outcomes (medium effect size) and child outcomes (small effect size; Nieuwboer et al., 2013). In this meta-analysis, it was concluded that online interventions have the potential to not only increase parental knowledge, but also to improve parental attitude, parenting abilities and behavior (Nieuwboer et al., 2013).

This study investigated the effectiveness of an online mindful parenting training for mothers with young children who experience parental stress. It utilized a randomized controlled design, with an intervention group and a waitlist control group. It was hypothesized that the online mindful parenting training would (1) decrease parental stress, (2) decrease over-reactive parenting discipline, (3) improve mindful parenting and self-compassion, (4) decrease maternal symptoms of anxiety and depression, and (5) decrease child behavior problems.

## Materials and Methods

### Participants

**FIGURE 1:**
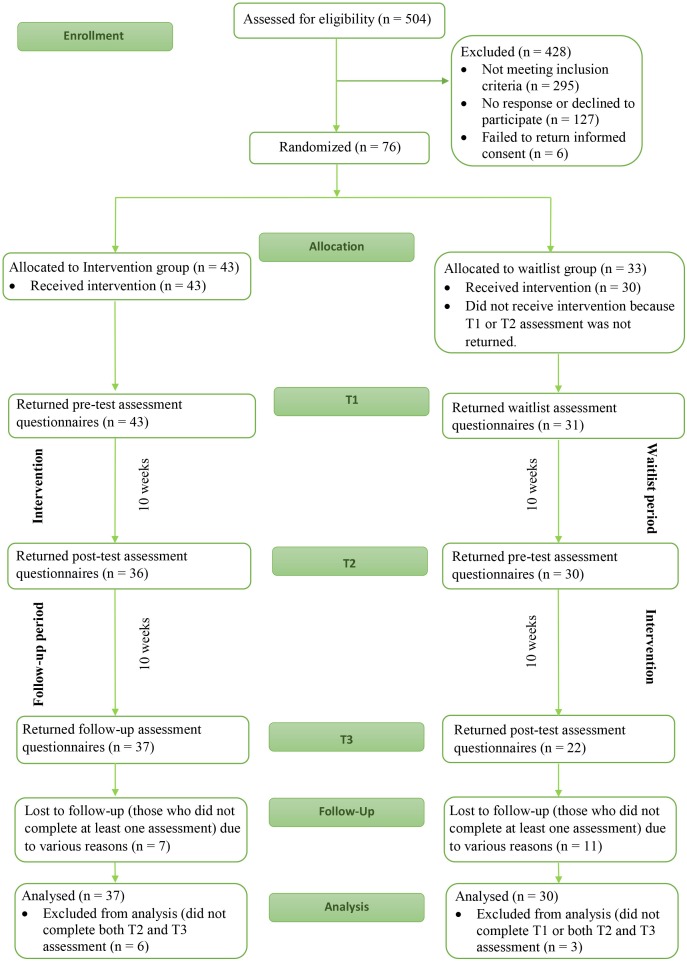
Flowchart for the participating women in the current study.

During the perinatal period, all participating mothers of the current study took part in a large longitudinal cohort study based in the Southern region of the Netherlands: the Holistic Approach to Pregnancy and the first Postpartum Year (HAPPY) study. A detailed protocol of the HAPPY study has previously been described (Truijens et al., 2014). Inclusion criteria for participation in the cohort study were: singleton pregnancy and a sufficient understanding of the Dutch language. Exclusion criteria were: chronic disease (e.g., diabetes, thyroid dysfunction), severe psychopathology (e.g., schizophrenia, borderline personality disorder, bipolar disorder) and very preterm childbirth (gestational age < 32 weeks). Following the HAPPY study, a subsample of approximately 500 mothers and their toddlers participated in the HAPPY follow study and were assessed at 2 and/or 3–3.5 years postpartum, and completed the Parental Stress Questionnaire (PSQ; Vermulst et al., 2012). Of 504 women who completed the PSQ, 209 (41%) showed an elevated level of parental stress (T-score ≥64) on at least one of three subscales related to parenting, namely (1) parent child relationship problems, (2) parenting problems, and (3) parental role restriction. These 209 mothers with elevated levels of parental stress were eligible for the current study, and were invited by e-mail. Of the invited mothers, 127 (61%) did not respond to, or declined the invitation. Of the 82 mothers that accepted the invitation, six mothers (7%) failed to return informed consent. Thus, a total of 76 women were included in the current study and were randomized to either an intervention group (*n* = 43) or a waitlist group (*n* = 33) (see Figure 1).

Sample size calculation was based on an expected medium effect size improvement in parental stress (Spijkerman et al., 2016). To achieve a power of 80% to find a significant interaction between-within subjects, with 10% alpha, 100 participants were needed (50 per treatment group). It was predicted that these numbers could be included, because we expected the percentage of eligible mothers that chose to participate in the study to be higher (50%). The fact that both groups were smaller than proposed may have negatively influenced the power of the current study.

### Procedure and Design

The current study is a randomized waitlist controlled trial design. The trial is registered in the Dutch Trial Register (NTR7401) and was approved of by the Ethics Committee of the University of Amsterdam. Written informed consent was obtained from all participants included in the study. Participants were randomized before completing T1, but were only informed about the group they were allocated to after completing T1. For the intervention group, T1 served as a pretest assessment, while T1 served as a waitlist assessment for the waitlist group. This was followed by an immediate intervention period for the intervention group, and a waitlist period for the waitlist group. The intervention group participants were given 10 weeks to complete the 8-week intervention, taking into consideration that certain circumstances could result in extra time being needed (e.g., vacation or sickness) and therefore allowing for participants to complete the intervention. The waitlist period for the waitlist control group also lasted 10 weeks. This 10-week period was followed by T2: posttest assessment for the intervention group and pretest assessments for the waitlist control group. This was followed by another 10-week period. These 10 weeks served as a follow-up period for the intervention group, but also as the intervention period for the waitlist group. The last assessment for both groups was T3: follow-up assessment for the intervention group and posttest assessment for the waitlist group (see Figure 1).

Assessments consisted of questionnaires about maternal functioning that were completed online by participating mothers, and questionnaires on child functioning that were completed online by both the participating mothers and the non-participating parents (which was another mother in one case, and the father in all other cases). The post-test assessment (which was T2 for the intervention group and T3 for the waitlist group) also included questions about the number of sessions completed, and time spent meditating.

### Intervention

The online mindful parenting training for mothers with toddlers is based on the Mindful Parenting training developed by Bögels and Restifo (2013) and the Mindful with your toddler training (Potharst et al., in press b). Modifications were made to cater to the online format as well as to age specific themes. The training was developed by a mindful parenting specialist (EP) and an online-intervention specialist (VS). All participants created a password-protected personal account on the intervention website^1^. The training consists of 8 weekly online sessions. Each session consists of the following format: (1) a weekly theme, introduced by a mindfulness trainer in video format, (2) formal meditations (body scan, sitting meditations, walking meditation, mindful movement) (3) other exercises, such as visualization exercises, (4) information about how to deal with difficulties during practice, (5) psychoeducation about a mindful parenting theme relevant for parents with a toddler, and (6) exercises for daily home practice, including formal meditation (of about 10 to a maximum of 20 min), informal meditation and mindful parenting practice. After completing an exercise, mothers were invited to write about their experiences during the practice. During the training, parents learn to become aware of their own experience, also when interacting with their child. They are also taught to reflect on the experience and needs of the child. Parents additionally learn to recognize signals of stress in themselves, and to apply short mindfulness exercises in moments of stress. Mothers practiced self-care by being kinder to themselves. The training is fully self-directed and does not involve contact with a mindful parenting trainer or with other parents. Session length ranges from about 35 to 50 min. An outline of the mindful parenting training is displayed in Table 1.

**Table 1:** Outline of the 8 sessions of the online mindful parenting training.

*1 Automatic pilot* Exercises: Intention meditation, visualization exercise about automatic stress reaction, body scan Psychoeducation about automatic pilot and mindful parenting, and seven attitudinal foundations
*2 Beginner’s mind* Exercises: Sitting meditation with attention to breathing, visualization exercise about the way one tends to relate and react to oneself Psychoeducation about beginner’s mind, breathing
*3 At home in your body* Exercises: Mindful movement, 3-min breathing space Psychoeducation about the body, and supporting the autonomy of a child
*4 Responsive versus reactive parenting* Exercises: Sitting meditation with attention breathing and the body, and visualization exercise on the use of the 3-min breathing space in stressful parenting situations Psychoeducation about responsive versus reactive parenting
*5 Self-compassion* Exercises: Sitting meditation with attention for sounds and thoughts, reflection exercise about avoidance, self-compassion meditation Psychoeducation about self-compassion
*6 Conflict and repair* Exercises: Walking meditation, visualization exercise on conflict and repair with the child Psychoeducation about conflict and repair and stress and perspective taking
*7 Boundaries and taking care of yourself* Exercises: Sitting meditation with open attention, visualization exercise on boundaries, exercise on own needs Psychoeducation about boundaries and taking care of the self
*8 Mindful parenting – day by day* Exercises: mountain meditation, visualization exercise about looking back and looking ahead, making of a meditation plan. Psychoeducation about continuing with mindful parenting after the training


### Measures

#### Primary Outcome Measure: Parental Stress

Parental stress experienced by the participating women was measured using the Parental Stress Questionnaire (in Dutch: Opvoedingsbelastingvragenlijst) (PSQ, Vermulst et al., 2012), which is based on the Parenting Stress Index (Abidin, 1983). This questionnaire for parents of chidren aged 0 to 18, consists of 34 items that are rated on a scale from 1 to 4 (1, not true; 2, somewhat true; 3, quite true; 4, very true). The PSQ has 5 subscales: (1) *parent-child relationship problems*, (2) *parenting problems*, (3) *parental role restriction*, (4) *depressive mood*, and (5) *physical health problems*. This study used only the first three subscales, which are related to parenting. Examples of items of the first three subscales are, respectively: (1) “My child is a source of enjoyment,” (2) “I am in charge when I am with my child,” and (3) “I have less contact with friends than I used to because of my child.” In order to interpret the level of parental stress experienced, subscale scores were converted into T-scores conform the norms of the child’s age (e.g., 0 to 3 years). The PSQ and its subscales have good reliability and validity (Vermulst et al., 2012; Veerman et al., 2014). The Cronbach’s alpha in the current study were: 0.84 for parent-child relationship problems, 0.85 for parenting problems and 0.82 for parental role restriction.

#### Secondary Outcome Measures: Maternal Functioning

##### Over-reactive parenting discipline

Mothers were asked to complete the 10-item overreactivity subscale of the Parenting Scale (Arnold et al., 1993). The concept of this subscale refers to a parenting discipline that is harsh and authoritative. For each item, participants are provided with two opposite statements and are asked to indicate how they react to specific situations regarding their child, on a spectrum scaled 1 to 7. For example, “when there is a problem with my child,” one end of the spectrum is: “things build up and I do things I do not mean to do,” and the other: “things do not get out of hand.” Total scores range from 10 to 70, with higher total scores indicating a more inadequate parenting discipline. The parenting scale has adequate reliability and validity (Arnold et al., 1993). The Cronbach’s alpha for the overreactivity subscale in the current study was 0.81.

##### Mindful parenting

The Dutch 10-item (original) version of the Interpersonal Mindfulness in Parenting Scale (IM-P; Duncan, 2007; De Bruin et al., 2014) was used to measure mindful parenting. The Cronbach’s alpha for this scale in the current study was 0.49. Considering the weak internal consistency, we did not analyze the IM-P in the current study.

##### Self-compassion

The 3-item version of the Self-Compassion Scale was administered to assess self-compassion (SCS-3, Raes and Neff, unpublished manuscript). The SCS-3 is derived from the Self-Compassion Scale and its short-form (SCS (-SF), Neff K. D., 2003; Raes et al., 2011). The three items of this scale are: “I try to see my failings as part of the human condition,” “When I am feeling down I tend to obsess and fixate on everything that is wrong” (reverse coded), and “I am intolerant and impatient toward those aspects of my personality I do not like” (reverse coded). These items represent three domains of the self-compassion scale, namely *common humanity, mindfulness*, and *self-kindness*. On a scale of 1 to 5 (1, almost never; 5, almost always), participants were asked to express how frequently they acted as specified in the given statement. The range of the total score is 3 to 15, with higher scores indicating greater levels of self-compassion. The Cronbach’s alpha for this scale in the current study was 0.81.

##### Symptoms of depression and anxiety

To assess symptoms of depression and anxiety, a short screening tool was used: the Patient Health Questionnaire-4 (PHQ-4, Kroenke et al., 2009). The PHQ-4 is a four-item scale that was formed by merging the General Anxiety Disorder-2 (GAD-2, Kroenke et al., 2007) and the Patient Health Questionaire-2 (PHQ-2, Kroenke et al., 2003). For each item, women were asked to indicate how frequently they had faced the described statement over the past 2 weeks on a 4-point Likert scale (0, not at all; 1, several days; 2, more than half the days; and 3, nearly every day). Total scores range from 0 to 12, with higher score indicating more symptomatology. Examples of items are: “feeling nervous, anxious or on edge” and “little interest or pleasure in doing things.” The PHQ-4 is a reliable and valid instrument (Kroenke et al., 2009; Löwe et al., 2010). The Cronbach’s alpha for the total PHQ-4 score in the current study was 0.80.

#### Secondary Outcome Measures: Child Behavior

##### Child aggressive behavior and emotional reactivity

Both the participating mothers as well as the non-participating parent evaluated problem behavior of their toddler. Two subscales of the Dutch Child Behavior Checklist for children aged 1½ to 5 (CBCL 1½ – 5, Achenbach and Rescorla, 2000) were completed: (1) *Aggressive Behavior* and (2) *Emotionally Reactive*. For each item, both parents specified to which extent it is applicable to how the child has been in the past 2 months. Items are rated on a scale of 0 to 2 (0, not at all; 1, sometimes; 2, often). Total scores were calculated for each subscale and were converted into T-scores. Examples of items for each subscale, respectively, are: (1) “punishment does not change his/her behavior” and (2) “shows panic for no good reason.” In the current study, the Cronbach’s alpha for each subscale of the CBCL completed by mother was 0.88 and 0.70, respectively, and for the non-participating parent the Cronbach’s alpha for each subscale was 0.80 and 0.78, respectively.

### Data Analyses

All primary and secondary outcome measures were normally distributed at T1, where skewness and kurtosis were between -1 and +1 (George and Mallery, 2014). The intervention and waitlist group were compared regarding sociodemographic variables, using independent *t*-tests and chi-square tests. Baseline differences between the groups on all outcomes (T1) were analyzed using independent *t*-tests. If differences between the groups in baseline maternal functioning were found, these differences were controlled for in subsequent analyses of mother-rated outcome measures.

Intervention group changes over time and differences in changes over time between the intervention and waitlist group on all outcome measures were analyzed using multilevel regression models (mixed models). The structure of the multilevel models consisted of repeated measurements of time (fixed effects, level 1), nested in participants (level 2). Measurements were dummy coded with T1 scores as reference. Besides measurement occasions, the variables group [intervention (used as a reference) or waitlist] and PHQ-score at T1 (control variable) were added. Data were analyzed to assess whether change before and after the intervention was different than change before and after a waitlist period. To test whether this difference was present, we added interaction variable group^∗^T2 to the models. The interaction variable group^∗^T3 was added to test whether change between T1 and T3 was the same for both groups, as at T3, the waitlist group had also received the intervention. Scores on all outcomes were standardized across assessments, so that estimates of regression coefficients for dichotomous explanatory variables (measurement occasion, group, interaction between measurement occasion and group) can be interpreted similarly to Cohen’s *d* effect sizes (0.2 small, 0.5 medium, 0.8 large; Cohen, 1988), and estimates of regression coefficients for continues explanatory variables (PHQ score at T1) can be interpreted similarly to Pearson *r* effect sizes (0.1 small, 0.3 medium, 0.5 large; Cohen, 1988). The intercept was a random effect in all models. For multilevel analyses all cases are included, including those with missing data (Bagiella et al., 2000). Therefore, all participants that completed T1 and at least one more measurement (T2 and/or T3) were included in the analyses. Data analysis was performed according to the intention to treat analysis design. Because one-sided tests were used, results were considered significant if *p* < 0.10. For the primary outcome measures and for secondary outcome measures that showed significant within- or between-group differences, figures were made to give more insight into the direction of the differences.

Dose-response relationship was additionally assessed. It was checked whether the number of sessions completed and the number of minutes spent meditating per week were associated with the degree to which improvement between pretest and posttest was reported. This was assessed for all outcome measures that showed significant within- and/or between-group differences. The number of sessions and the number of minutes spent meditating per week were not normally distributed. Therefore, Spearman correlations were used. Improvement between pretest and posttest was calculated by subtracting posttest scores from pretest scores (T1 minus T2 scores for the intervention group, and T2 minus T3 scores for the waitlist group).

## Results

### Response Rate and Adherence to Intervention

Of the 43 mothers that were randomized to the intervention group, 43 (100%) completed T1, 36 (84%) completed T2, and 37 (86%) completed T3. Of the 33 mothers that were randomized to the waitlist control group, 31 (94%) completed T1, 30 (91%) completed T2, and 22 (67%) completed T3. Of the intervention group, six participants (14%) were excluded because of missing both the T2 and T3 measurement. Of the waitlist group, three (9%) participants were excluded because of missing all three measurements or missing both the T2 and T3 measurement. A total of 37 and 30 mothers were included in the analyses for the intervention and waitlist control group, respectively (See Figure 1). During the 10 weeks between pre- and posttest, participants in the intervention group completed an average of 3.8 sessions of the intervention (SD = 2.59, range 1–8 sessions), and participants in the waitlist control group an average of 3.8 sessions (SD = 2.60, range 1–8 sessions). Of the women who completed posttest assessment, five (13.9%) women in the intervention group (T2) and 4 (18.2%) women in the waitlist group (T3) completed the entire training Apart from following the sessions, participants were invited to practice formal meditation daily. Average time spent on meditating was 14.94 min per week in the intervention group (SD = 26.30, range 0–120 min), and 18.68 min per week in the waitlist control group (SD = 30.33, range 0–120 min). No significant difference in adherence to the sessions and the practice of formal meditation between the groups occurred.

### Differences Between the Groups at Baseline

The demographic characteristics of the participants are displayed in Table 2. No significant differences in demographic characteristics were found between the intervention and waitlist group. Mean scores (SD) on all primary outcome measures, secondary outcome measures: maternal wellbeing and secondary outcome measures: child behavior at T1, T2 and T3 (pretest, posttest and follow-up assessment for the intervention group and waitlist, pretest and posttest assessment for the waitlist group) are displayed in Tables 3–5, respectively. It was checked whether the intervention group differed from the waitlist group on any of the outcome measures at T1; this was the case for symptoms of depression and anxiety (PHQ-4). The intervention group reporting significantly more symptoms of depression and anxiety than the waitlist control group [*n* = 67, *t* (65) = 2.28, *p* = 0.026, 95% *CI* (0.18, 2.73), *d* = 0.57]. Therefore, it was decided to control for symptoms of depression and anxiety in subsequent analyses of mother-rated outcome measures.

**Table 2:** Demographic characteristics of the participating mothers (*n* = 67).

	Intervention group (*n* = 37)				Waitlist group (*n* = 30)					
				
	N	%	Mean (SD)	Range	N	%	Mean (SD)	Range	*t*	χ^2^
**Demographics**										
Age			35.8 (3.6)	26–45			36.7 (4.2)	30–45	-0.89	
Level of education										
Low	2	5.4			0	0				1.62
Medium	6	16.2			5	17.2				
High	29	78.4			24	82.8				
Paid job	31	83.8			27	90.0				0.55
Living with partner	37	100			29	96.7				1.25
**Child characteristics**										
Age			3.5 (0.23)	3.1–4.2			3.5 (0.31)	3.2–4.7	-0.39	
Gender										
Girl	21	58.3			16	53.3				0.17
Boy	15	41.7			14	46.7				
Number of children in household										
One	7	18.9			3	10.0				4.15
Two	18	48.6			21	70.0				
Three	7	18.9			5	16.7				
Four or more	5	13.5			1	3.3				


*SD, standard deviation; level of education; low, primary education or secondary pre-vocational education; medium, secondary education or vocational education; high, Bachelor or Master’s degree. ^∗^*p* < 0.05.*

**Table 3:** Mean and standard deviations for the primary outcome measure regarding parental stress at each measurement point.

	Intervention group		Waitlist group	
		
	*n*	*M (SD)*	*n*	*M (SD)*
**Primary outcome measure: Parental stress (PSQ)**				
- Parent-child relationship problems				
Waitlist	–	–	30	10.1 (2.6)
Pretest	37	10.5 (2.3)	30	10.0 (2.8)
Posttest	36	10.8 (2.6)	22	9.6 (2.1)
Follow-up	37	10.2 (2.7)	–	–
- Parenting problems				
Waitlist	–	–	30	14.2 (2.9)
Pretest	37	15.1 (3.2)	30	14.2 (3.3)
Posttest	36	14.5 (2.8)	22	13.5 (2.5)
Follow-up	37	14.7 (3.5)	–	–
- Parental role restriction				
Waitlist	–	–	30	13.6 (3.5)
Pretest	37	12.5 (2.6)	30	13.2 (4.0)
Posttest	36	12.6 (3.0)	22	13.5 (3.6)
Follow-up	37	11.7 (2.5)	–	–


*M, mean; SD, standard deviation; PSQ, parental stress questionnaire.*

**Table 4:** Mean and standard deviations for secondary outcome measures regarding maternal functioning at each measurement point.

	Intervention group		Waitlist group	
		
	*n*	*M (SD)*	*n*	*M (SD)*
**Secondary outcome measures: Maternal functioning**				
Over-reactive parenting discipline (PS)							
Waitlist	–	–	30	30.6 (8.1)
Pretest	37	31.7 (9.24)	30	31.8 (8.1)
Posttest	35	29.6 (8.20)	22	26.7 (6.1)
Follow-up	37	28.7 (7.47)	–	–
**Self-compassion (SCS-3)**				
Waitlist	–	–	30	11.5 (3.9)
Pretest	37	10.5 (4.15)	30	12.2 (4.2)
Posttest	36	12.7 (3.91)	22	13.1 (3.3)
Follow-up	37	12.1 (3.82)	–	–
**Symptoms of depression and anxiety (PHQ-4)**				
Waitlist	–	–	30	2.6 (2.2)
Pretest	37	4.05 (2.85)	30	3.3 (3.1)
Posttest	36	3.11 (3.18)	22	2.5 (2.1)
Follow-up	37	2.43 (2.59)	–	–


*M, mean; SD, standard deviation; PS, parenting scale; SCS-3, 3-item self-compassion scale; PHQ-4, Patient Health Questionnaire – 4.*

**Table 5:** Mean and standard deviations for secondary outcome measures regarding child behavior at each measurement point.

	Intervention group		Waitlist group	
		
	*n*	*M (SD)*	*n*	*M (SD)*
**Secondary outcome measure: Child behavior (CBCL)**				
- Child aggressive behavior assessed by the participating mother				
Waitlist	–	–	30	14.5 (5.9)
Pretest	37	16.0 (7.2)	30	14.3 (6.2)
Posttest	36	13.6 (5.9)	22	13.5 (6.4)
Follow-up	37	13.5 (6.7)	–	–
- Child emotional reactivity assessed by the participating mother				
Waitlist	–	–	30	4.77 (2.7)
Pretest	37	5.03 (3.0)	30	5.23 (3.5)
Posttest	36	4.31 (2.8)	22	4.73 (2.9)
Follow-up	37	4.46 (3.5)	–	–
- Child aggressive behavior assessed by the non-participating parent				
Waitlist	–	–	23	15.2 (4.6)
Pretest	37	14.3 (5.4)	23	14.2 (5.3)
Posttest	34	14.6 (6.4)	22	13.6 (6.0)
Follow-up	28	13.7 (6.5)	–	–
- Child emotional reactivity assessed by the non-participating parent				
Waitlist	–	–	23	4.61 (2.3)
Pretest	36	5.64 (3.4)	24	4.08 (2.9)
Posttest	34	6.00 (3.5)	22	4.18 (2.5)
Follow-up	28	5.75 (3.7)	–	–


*M, mean; SD, standard deviation; CBCL, child behavior checklist.*

### Intervention Effects on Outcome Measures

Results of multilevel models of treatment outcome predicted by measurement occasion are shown in Table 6 for the primary outcome measure parental stress, in Table 7 for secondary outcome measures regarding maternal functioning, and in Table 8 for secondary outcome measures regarding child behavior.

**Table 6:** Primary outcome measure (parental stress): Standardized parameter estimates (and standard errors), *t* and *p* values, and 95% confidence intervals of multilevel models of intervention outcome predicted by measurement point (T2 and T3, deviations from T1), group [intervention (reference) and waitlist control group], control variable (PHQ-4 score at T1), and interaction variables (group by T2 and T3).

						Interaction	
	
	Intercept	T2	T3	Group	PHQ-4 at T1	Group x T2	Group x T3
**Primary outcome measure: Parental stress (PSQ)**							
- Parent-child relationship problems							
*β* (SE)	-0.16 (0.23)	0.15 (0.15)	-0.13 (0.12)	-0.06 (0.24)	0.07 (0.04)	-0.20 (0.22)	0.01 (0.19)
*t*	-0.72	1.03	-1.09	-0.23	1.64	-0.94	0.07
*p*	0.476	0.305	0.280	0.816	0.106	0.352	0.946
95% CI	(-0.61, 0.29)	(-0.14, 0.44)	(-0.36, 0.11)	(-0.53, 0.42)	(-0.01, 0.15)	(-0.64, 0.23)	(-0.36, 0.38)
- Parenting problems							
*β* (SE)	0.15 (0.24)	-0.14 (0.11)	-0.12 (0.13)	-0.27 (0.25)	0.01 (0.04)	0.14 (0.17)	-0.03 (0.20)
*t*	0.64	-1.23	-0.99	-1.06	0.32	0.83	-0.14
*p*	0.524	0.222	0.327	0.291	0.747	0.410	0.887
95% CI	(-0.32, 0.63)	(-0.36, 0.09)	(-0.37, 0.13)	(-0.77, 0.23)	(-0.07, 0.10)	(-0.19, 0.47)	(-0.43, 0.37)
- Parental role restriction							
*β* (SE)	-0.72 (0.21)	0.02 (0.11)	-0.24 (0.11)	0.59 (0.22)	0.15 (0.04)	-0.16 (0.16)	0.02 (0.17)
*t*	-3.45^∗∗^	0.21	-2.21^∗^	2.71^∗∗^	4.13^∗∗∗^	-1.00	0.13
*p*	0.001	0.837	0.031	0.009	<0.001	0.321	0.895
95% CI	(-1.13, -0.30)	(-0.19, 0.24)	(-0.45, -0.02)	(0.16, 1.03)	(-0.08,0.23)	(-0.47,0.16)	(-0.32,0.37)


*^†^*p* < 0.10, ^∗^*p* < 0.05, ^∗∗^*p* < 0.01, ^∗∗∗^*p* < 0.001, SE, standard error; *β*, Standardized parameter estimate can be interpreted similar to Cohen’s *d* in case of dichotomous variables, and as Pearson *r* in case of continuous variables; CI, confidence interval; PSQ, parenting stress questionnaire; PHQ-4, Patient Health Questionnaire – 4.*

**Table 7:** Secondary outcome measures (maternal functioning): Standardized parameter estimates (and standard errors), *t* and *p* values, and 95% confidence intervals of multilevel models of intervention outcome predicted by measurement point (T2 and T3, deviations from T1), group [intervention (reference) and waitlist control group], control variable (PHQ-4 score at T1), and interaction variables (group by T2 and T3).

						Interaction	
	
	Intercept	T2	T3	Group	PHQ-4 at T1	Group x T2	Group x T3
**Secondary outcome measures: Maternal functioning**							
**Over-reactive parenting discipline (PS)**							
*β* (SE)	-0.05 (0.24)	-0.26 (0.11)	-0.37 (0.11)	-0.05 (0.26)	0.06 (0.04)	0.41 (0.17)	0.02 (0.18)
*t*	-0.20	-2.28^∗^	-3.28^∗∗^	-0.17	1.57	2.45^∗^	0.85
*p*	0.845	0.026	0.002	0.862	0.123	0.017	0.932
95% CI	(-0.52, 0.42)	(-0.49, -0.03)	(-0.59, -0.14)	(-0.57, 0.48)	(-0.02, 0.14)	(0.08, 0.75)	(-0.34, 0.37)
**Self-compassion (SCS-3)**							
*β* (SE)	0.41 (0.20)	0.57 (0.14)	0.40 (0.14)	-0.01 (0.22)	-0.19 (0.03)	-0.40 (0.20)	0.08 (0.21)
*t*	2.03^∗^	4.20^∗∗∗^	2.99^∗∗^	-0.06	-5.53^∗∗∗^	-1.95^†^	0.40
*p*	0.046	<0.001	0.004	0.953	<0.001	0.056	0.695
95% CI	(0.01, 0.81)	(0.30, 0.85)	(0.13, 0.68)	(-0.46, 0.43)	(-0.26, -0.12)	(-0.80, 0.01)	(-0.34, 0.50)
**Symptoms of depression and anxiety (PHQ-4)**							
*β* (SE)	0.38 (0.15)	-0.36 (0.16)	-0.58 (0.15)	-0.52 (0.23)	–	0.59 (0.24)	0.50 (0.24)
*t*	2.50^∗^	-2.24^∗^	-3.82^∗∗∗^	-2.32^∗^	–	2.53^∗^	2.05^∗^
*p*	0.015	0.028	<0.001	0.024	–	0.014	0.044
95% CI	(0.08, 0.68)	(-0.67, -0.04)	(-0.89, -0.28)	(-0.97, -0.07)	–	(0.13, 1.06)	(0.01, 0.98)


*^†^*p* < 0.10, ^∗^*p* < 0.05, ^∗∗^*p* < 0.01, ^∗∗∗^*p* < 0.001; SE, standard error; *β*, Standardized parameter estimate can be interpreted similar to Cohen’s *d* in case of dichotomous variables, and as Pearson *r* in case of continuous variables; CI, confidence interval; PS, Parenting Scale; SCS-3, 3-item self-compassion scale; PHQ-4, Patient Health Questionnaire – 4.*

**Table 8:** Secondary outcome measures (child behavior): Standardized parameter estimates (and standard errors), *t* and *p* values, and 95% confidence intervals of multilevel models of intervention outcome predicted by measurement point (T2 and T3, deviations from T1), group [intervention (reference) and waitlist control group], control variable (PHQ-4 score at T1), and interaction variables (group by T2 and T3).

						Interaction	
	
	Intercept	T2	T3	Group	PHQ-4 at T1	Group x T2	Group x T3
**Secondary outcome measure: Child behavior (CBCL)**							
- Child aggressive behavior assessed by the participating mother							
*β* (SE)	0.40 (0.24)	-0.33 (0.11)	-0.38 (0.11)	-0.28 (0.26)	-0.04 (0.04)	0.29 (0.17)	0.30 (0.18)
*t*	1.68^†^	-2.90^∗∗^	-3.34^∗∗^	-1.08	-0.86	1.74^†^	1.63
*p*	0.097	0.005	0.001	0.284	0.392	0.087	0.108
95% CI	(-0.07, 0.88)	(-0.56, -10)	(-0.61, -0.15)	(-0.79, 0.23)	(-0.12, 0.05)	(-0.04, 0.63)	(-0.07, 0.66)
- Child emotional reactivity assessed by the participating mother							
*β* (SE)	-0.11 (0.22)	-0.20 (0.13)	-0.19 (0.13)	-0.01 (0.23)	0.05 (0.04)	0.35 (0.19)	0.25 (0.21)
*t*	-0.49	-1.61	-1.40	-0.04	1.25	1.90^†^	1.72
*p*	0.627	0.113	0.167	0.967	0.215	0.062	0.246
95% CI	(-0.56, 0.34)	(-0.45, 0.05)	(-0.45, 0.08)	(-0.47, 0.45)	(-0.03, 0.13)	(-0.02, 0.73)	(-0.17, 0.67)
- Child aggressive behavior assessed by the non-participating parent							
*β* (SE)	0.01 (0.14)	0.07 (0.14)	-0.17 (0.14)	0.14 (0.23)	–	-0.28 (0.22)	-0.10 (0.22)
*t*	0.061	0.51	-1.22	0.60	–	-1.24	-0.43
*p*	0.951	0.613	0.229	0.552	–	0.219	0.668
95% CI	(-0.28, 0.30)	(-0.21, 0.35)	(-0.46, 0.11)	(-0.32, 0.59)	–	(-0.72, 0.17)	(-0.54, 0.35)
- Child emotional reactivity assessed by the non-participating parent							
*β* (SE)	0.14 (0.15)	0.10 (0.15)	-0.05 (0.16)	-0.33 (0.24)	–	-0.30 (0.23)	-0.09 (0.25)
*t*	0.94	0.70	-0.28	-1.36	–	-1.28	-0.35
*p*	0.350	0.490	0.778	0.179	–	0.205	0.730
95% CI	(-0.16, 0.44)	(-0.19, 0.40)	(-0.37, 0.28)	(-0.80, 0.15)	–	(-0.77, 0.17)	(-0.59, 0.42)


*^†^*p* < 0.10, ^∗^*p* < 0.05, ^∗∗^*p* < 0.01, ^∗∗∗^*p* < 0.001; SE, standard error; *β*, Standardized parameter estimate can be interpreted similar to Cohen’s *d* in case of dichotomous variables, and as Pearson *r* in case of continuous variables; CI, confidence interval; PHQ-4, Patient Health Questionnaire – 4; CBCL, child behavior checklist.*

### Primary Outcome Measure: Parental Stress

**FIGURE 2:**
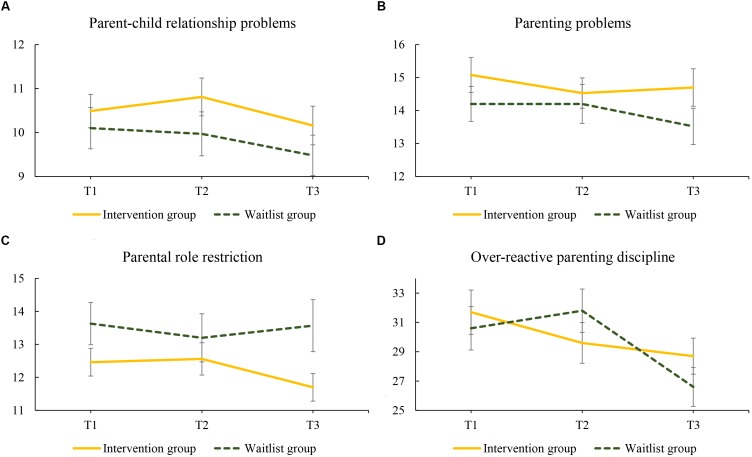
Changes in mean scores regarding **(A)** parent-child relationship problems, **(B)** parenting problems, **(C)** parental role restriction, and **(D)** over-reactive parenting discipline for women in the intervention and waitlist group over the 20-week study period: T1 to T2 (0–10 weeks) and T2 to T3 (10–20 weeks). Intervention group assessments: T1, pretest; T2, posttest; T3, follow-up; Waitlist group assessment: T1, waitlist; T2, pretest; T3 posttest.

Interaction between group and T2 was non-significant for all three subscales of the PSQ, showing that there was no difference in improvement between intervention and waitlist (see Figure 2A–C). Regarding within-group effects: a significant delayed effect (at T3, follow-up) at a 95% level was found on subscale parental role restriction for the intervention group (small effect size) (see Table 6).

### Secondary Outcome Measures: Maternal Functioning

Regarding over-reactive parenting discipline, a significant interaction (95% level) between group and T2 showed that effects of intervention and waitlist differed, in favor of the intervention group (see Figure 2D). The absence of a significant interaction between group and T3 showed that after the waitlist group had also received the intervention (posttest), their improvement in over-reactive parenting discipline was similar to the improvement of the intervention group at follow-up. Looking at within-group differences for the intervention group, a significant improvement (95% level) in over-reactive parenting discipline was found at both T2 (posttest) and T3 (follow-up, small effect sizes) (see Table 7).

**FIGURE 3:**
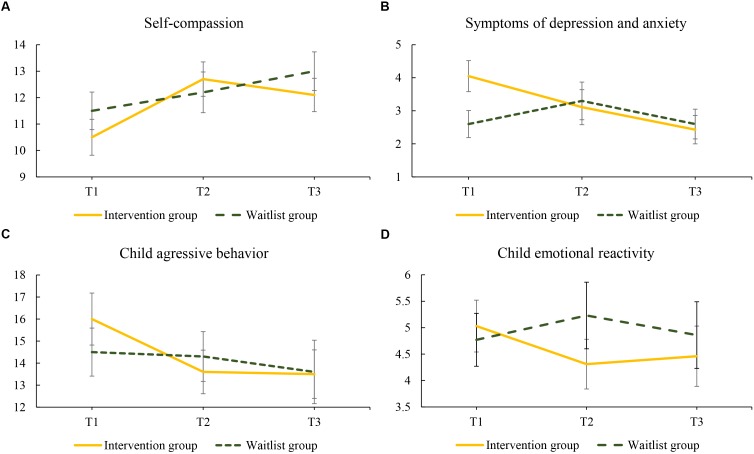
Changes in mean scores regarding **(A)** self-compassion, **(B)** symptoms of depression and anxiety, **(C)** child aggressive behavior, and **(D)** child emotional reactivity for women in the intervention and waitlist group over the 20-week study period: T1 to T2 (0–10 weeks) and T2 to T3 (10–20 weeks). Intervention group assessments: T1, pretest; T2, posttest; T3, follow-up; Waitlist group assessment: T1, waitlist; T2, pretest; T3 posttest.

With regard to self-compassion, the interaction between group and T2 was significant at a 90% level, showing that the effect of intervention was larger than the effect of waitlist (small effect size, see Figure 3A). The absence of a significant interaction between group and T3 showed that after the waitlist group had also received the intervention (posttest), their improvement in self-compassion was similar to the improvement of the intervention group at follow-up. Looking at within-group differences for the intervention group, an improvement, significant at 95% level, in self-compassion was found at both T2 (posttest, medium effect size) and T3 (follow-up, small effect size) (see Table 7).

For symptoms of depression and anxiety, an interaction between group and T2, that was significant at 95% level, also showed differential effects of intervention and waitlist in favor of the intervention group (medium effect size, see Figure 3B). Interaction between group and T3 was also significant (95% level), showing that improvement of the intervention group was of larger effect size at follow-up, than it was for the waitlist group after they had received the intervention (medium effect size). For the intervention group, significant within-group improvements (95% level) in symptoms of depression and anxiety were shown at T2 (posttest, small effect size) and T3 (follow-up, medium effect size) (see Table 7).

### Secondary Outcome Measures: Child Behavior

Child behavior was rated by both parents. The interaction between group and T2 was significant at a 90% level in multilevel models predicting both mother-rated child aggressive behavior and child emotional reactivity, showing that there was a difference in effect for the intervention and waitlist control group (small effect sizes, see Figure 3C,D). The absence of a significant interaction between group and T3 for mother-rated child aggressive behavior and emotional reactivity showed that after the waitlist group had also received the intervention (posttest), the improvement in mother-reported child behavior was similar to the improvement of the intervention group at follow-up. Looking at within-group change, improvement at both T2 (posttest) and T3 (follow-up, small effect sizes) was found for child aggressive behavior, but not for child emotional reactivity (see Table 8).

For child aggressive behavior and emotional reactivity as assessed by the non-participating parent, no significant interactions between group and measurement occasion were found, as well as no significant within-group effects (see Table 8).

### Dose-Response Relationship

Spearman correlations were calculated to study the dose-response relationship. A higher number of completed sessions was significantly associated with greater improvement on three outcome measures, namely parental role restriction (*r*_s_ = 0.26, *p* = 0.047), self-compassion (*r*_s_ = -0.43, *p* = 0.001), and symptoms of depression and anxiety (*r*_s_ = 0.26, *p* = 0.053). The number of minutes spent meditating was not associated with improvement on any of the outcome measures.

## Discussion

This study evaluated the effectiveness of an 8-week online mindful parenting training for mothers with young children who experience parental stress. A randomized controlled study design was utilized, with an intervention and a waitlist control group. The intervention group received the intervention during the first 10 weeks of the study, while the waitlist control group received the intervention during the second 10 weeks of the study. The online mindful parenting intervention was shown to be more effective than a waitlist period with regard to symptoms of depression and anxiety (medium effect size difference between the groups), over-reactive parenting discipline, self-compassion, and child emotional reactivity (small effect size difference). A significant within-group effect was found for the primary outcome: parental stress, with regard to the parental role restriction subscale. The intervention group showed a delayed improvement (small effect size) on this subscale. A within-group difference (small effect size) was also found for child aggressive behavior at both post-test and follow-up for the intervention group. No significant improvement was found on child outcomes for the non-participating parent.

With regard to the primary outcome measure parental stress, a delayed effect was found in the intervention group on the domain of parental role restriction, that is, only at follow-up (within-group effect of small effect size). During the toddler period, the child is dependent on parental presence and co-regulation, which mothers may experience as a constraint on their personal life with respect to activities that they enjoy for themselves. Possibly, by doing the training and reflecting on their own feelings, parents become more aware of their own needs. By taking the time to do the training and completing the daily exercises, they may realize that taking time for themselves is possible, which may help them to arrange activities for themselves. Alternatively, mothers may be able to accept the situation more than they did before, or they may be able to adjust their expectations. Accordingly, enjoying things for themselves, even if only for a short period of time and/or in presence of the child may seem more possible. An immediate improvement in parental role restriction was found in the waitlist group after the training. Possibly, the completion of the questionnaires twice before being able to start the training helped them prepare and profit more optimally from the training. Furthermore, the waiting period may have provided time to plan ahead and prepare for the start of the training more thoroughly.

Of the different parental stress domains, only parental role restriction improved over time, while parent-child relationship problems and parenting problems did not. Duncan et al. (2009) presented a model of mindful parenting that aimed to explain how mindful parenting could improve parenting and parent-child interactions. It is therefore surprising that especially these two domains of parental stress did not improve. Earlier studies that examined the effectiveness of mindful parenting group training have found improvements in parenting problems and the parent-child relationship (Bögels et al., 2014; Emerson et al., in press; Potharst et al., in press b). Also, in a study comparing mindful parenting training in clinical versus non-clinical settings, in which the same questionnaire was used as the one in the current study, improvements in problems with parenting and in the parent-child relationship were reported by the parents after the training in both settings (Potharst et al., in press a).

Two important differences between the face-to-face groups versus online training are the presence of a trainer/therapist and a peer group of parents, who can both in their own way offer support and empathy. Furthermore, they could provide a sense of the universality of parental difficulties, an understanding of the parenting situation, or advice on how to apply mindfulness in specific situations. A study that compared a self-directed versus therapist-assisted telehealth intervention for parents of children with autism spectrum disorder, found that contact with a therapist via video-conferencing could be of added value in online parenting programs (Ingersoll et al., 2016). Results showed that parents in both groups improved in observed parent fidelity, self-reported self-efficacy, stress, and positive perceptions of their child, but that the therapist-assisted group improved more regarding parent fidelity and positive perceptions of the child (Ingersoll et al., 2016). A meta-analysis of online parenting programs indicated that self-directed programs were more effective with regard to parental knowledge, while programs with professional support (coaching with an earpiece) had better results in terms of parental behavior (Nieuwboer et al., 2013). Furthermore, programs featuring both professional and peer support showed better results regarding attitudinal changes (Nieuwboer et al., 2013).

Another difference with the group training is that the online training was less intensive, contained a shorter session length, had lower adherence, and gave less support for carrying out home practice. In the current study, average time spent meditating (excluding time spent mediating during training sessions) was only 15 min per week by the intervention group, and 19 min per week by the waitlist control group in the period that they followed the training (between T2 and T3). In an afore mentioned study by Potharst et al. (in press b) in which improvements in problems with parenting and in the parent-child relationship were found with the same questionnaire that was used in the current study, an average meditation time of 2 h per week was reported. The limited meditation time in the current study may also have contributed to the lack of improvement in parenting and the parent-child relationship. A final difference to be mentioned is the methodological nature of this study. The current study was one of the first to utilize a randomized control design where participants were randomly assigned to an intervention or waitlist control group, while the other studies used pre- and posttest or quasi-experimental designs. Future studies should examine whether an e-health coach or possibilities to be in touch with other parents following the training can support the online format by assisting parents in specific problems regarding parenting or the parent child relationship.

A specific area of parenting that did improve significantly in the current study is over-reactive parenting discipline (small effect size interaction between group and T2). Possibly, this area of parenting was more susceptible to change in this online version of the mindful parenting training because it may depend more on insight rather than on the number of hours spent reflecting on experiences in mediations and inquiries, together with a group. The first session provided participants with psycho-education about automatic stress reactions (fight, flight, freeze) that also involves over-reactivity (fight). Thus, even parents that only completed the first (few) session(s), may have benefited from this psycho-education. Improvement in over-reactive parenting was indeed not associated with the number of minutes spent meditating per week, nor the number of completed sessions. Yet, the other outcome measures related to maternal functioning (for which improvement was shown) were related to the number of completed sessions.

The current study found that the online mindful parenting training yielded positive results regarding self-compassion (small effect size interaction at 90% significance level between group and T2). Mindful parenting teaches parents a certain framework that helps them relate differently, not only toward their child and the problems they experience (with their child), but also toward themselves. Mindful parenting can be used to find a balance between taking care of the children without losing sight of one’s own needs, learning how to better take care of oneself and be friendly toward oneself. An important element of self-compassion is a sense of common humanity (Neff K., 2003). Following a mindful parenting training in a group may enhance the experience of common humanity. Parents who normally feel alone in the difficulties they experience in parenthood, may feel reassured by the fact that other parents experience difficulties as well. The positive result regarding self-compassion in the current study shows that being with a group of people with similar problems is not a necessity to increase self-compassion.

Positive effects were also found for symptoms of depression and anxiety (medium effect size interaction between group and T2). A meta-analysis on the effectiveness of online mindfulness trainings also showed small but significant improvements in symptoms of depression and anxiety (Spijkerman et al., 2016). This meta-analysis found that the improvements in depression and anxiety in a population of healthy individuals were smaller in effect size compared to individuals with psychological symptoms, but these differences were not significant (Spijkerman et al., 2016). This seems to be in line with the results of the current study, where the intervention group also showed a greater improvement. The intervention group reported more symptoms of depression and anxiety than the waitlist group at baseline. At T3, after the waitlist control group had also received the intervention, improvement was still smaller than in the intervention group. The difference between the groups at baseline may have therefore enlarged the interaction effect between group and T2. The current study confirms that especially for parents with higher levels of depression and anxiety, a mindfulness training can be beneficial, even if the specific mindfulness training is primarily focused on parenting and parental stress.

As the participating mothers report some positive personal change after participating in the training, it is also of interest whether their children additionally profit from these (self-perceived) changes. Indeed, a significant interaction between group and T2 at 90% level was found for mother-rated child aggressive behavior (small effect sizes of interaction between group and T2). Effect sizes of within-group differences between pretest, posttest and follow-up in child aggressive behavior were similar to effect sizes on child externalizing psychopathology that were reported in a study on the effectiveness of mindful parenting group trainings in youth mental health care (Meppelink et al., 2016). These results were also comparable to the effect size of improvement in child behavior that was reported in a meta-analysis on the effectiveness of online parenting trainings (Nieuwboer et al., 2013).

The improvement in mother reported child aggressive behavior can be explained in three ways: (1) there was an actual improvement in aggressive behavior of the children, (2) there was an improvement in child aggressive behavior toward the mothers, (3) a change occurred in the experienced burden from their children’s behavior, and (4) a change for the mothers in the intervention group occurred in the way they perceived their child’s behavior due to the knowledge of being in the intervention group. To gain clarity on how to interpret mother-reported change in their children, partners were allowed to complete a questionnaire about their children’s behavior. The partners reported no change in their children’s aggressive behavior after the training. This seems to point at the second, third or fourth explanation for the change the mothers in the intervention group reported. When examining the mean scores over time, the post-test (T2) decrease in the intervention group seems to be larger than the post-test decrease (T3) in the waitlist group, which seems to point to the fourth explanation. However, child aggressive behavior scores were somewhat higher in the intervention group than in the waitlist group at baseline (T1), and were similar at T3, after both groups received the intervention. Possibly, a larger study, in which observational measures of parent-child interaction and child behavior are included, and in which mechanisms of change are studied, could provide more insight. Specifically, it could provide more clarity regarding the interpretation of self-reported change in children’s behavior after following an online mindful parenting training. However, the fact that the interpretation of the outcome on child behavior of the current study is unclear, does not imply that the outcome itself is trivial, as parental perception of child behavior can influence child development and child behavior (Bugental and Johnston, 2000).

Furthermore, regarding child emotional reactivity, a significant interaction between group and T2 at 90% level was found. High child emotional reactivity may be a result of emotion regulation problems (Cole et al., 2004; Morgan et al., 2014). The development of emotion regulatory abilities in children is dependent on child cognitive development and on child temperament, but also on parental emotion regulatory abilities (Rutherford et al., 2015). Training in mindfulness improves emotion regulation and decreases emotion regulation problems (Roemer et al., 2015). An example of this is the decrease in over-reactive parenting discipline in the current study. Therefore, online mindful parenting training may enhance the development of emotion regulatory abilities and decrease emotional reactivity in children.

Treatment fidelity was relatively low in the current study, and the variability in treatment fidelity was high. Average time spent meditating apart from the training sessions was about 15 min per week, with a range of 0 to 120 min. In an above mentioned study on a mindful parenting group training in a clinical and non-clinical setting, average meditation time was 2 h per week (Potharst et al., in press a). In other studies on online mindfulness interventions, participants also practiced more than in the current study. In a study on an online mindfulness training for employees, participants practiced on average 13 min per day or 1.5 h per week (Aikens et al., 2014). In two studies on preventative online mindfulness trainings, participants practiced on average 4 times a week for about 20 min (Morledge et al., 2013; Mak et al., 2015). In one of these studies, a weak but statistically significant correlation was found between the amount of practice and improvement on stress and mindfulness (Morledge et al., 2013). Possibly, the average amount of practice in the current study was too low to show such an association. The association between mindfulness practice and training outcome is, however, complex. In a randomized controlled trial on the effectiveness of mindfulness-based cognitive therapy (MBCT) group training for patients with recurrent depression, it was found that in general MBCT was not more effective in preventing a relapse than cognitive psychological education similar to what is taught in MBCT (Williams et al., 2014). Only for patients who scored above the median on level of childhood trauma, MBCT was shown to be more effective than psychoeducation without practice in mindfulness meditation (Williams et al., 2014).

Only 15.5% of the participants completed the training, and on average about four sessions were completed. In a meta-analysis on the effectiveness of online mindfulness interventions, five studies that reported the percentage of participants who completed the intervention were included, and these percentages ranged from about 40 to 90% (Spijkerman et al., 2016). In the study on a mindful parenting group training in a clinical and non-clinical setting adherence to the training was around 85% (Potharst et al., in press a). However, adherence to the training is not defined as completing all sessions in group trainings, as it is considered normal that participants miss one or a few sessions due to illness or vacation for example. The question is whether the online mindful parenting training in the current form is feasible for parents with parental stress. Possibly, the workload (e.g., daily homework, formal and informal meditation practice and mindful parenting practice) is too high for participants with a family with young children who already experience elevated levels of stress, given the lack of support or guidance by a trainer in the online format. It is, however, also possible that participants did not feel a need to follow more sessions than they did. This may have been the case for women who wanted to learn to be less over-reactive in their parenting, because over-reactive parenting discipline decreased significantly during the training, regardless of the number of sessions completed. Possibly, a shorter training fits better with the online format. In two studies, positive effects were reported of short (two session and/or 2 week trainings) on stress and symptoms of anxiety and depression (Glück and Maercker, 2011; Cavanagh et al., 2013). It is important to further study the feasibility and acceptability of the online mindful parenting training, and also focus on the facilitators and barriers for following the training.

The current study had both strengths and limitations. A major strength of the study was the utilization of a randomized design, and the fact that both the participating mothers and their partners participated in the study. One limitation is that at baseline, the intervention group reported more symptoms of depression and anxiety compared to the waitlist control group. This difference could not be explained by bias caused by participant’s knowledge of which group they were allocated to, as they were randomized after the completion of T1. It can also not be due to a greater drop-out rate by mothers with more symptoms of depression and anxiety in the waitlist group, which could have explained that they were in need of a short-term intervention or support. Of the three women that dropped out of the waitlist group, two never completed T1 and were therefore not informed about their allocation to waitlist control group, and the third participant had a very low score (2) on the PHQ-4. This difference between groups may have influenced the results, even though the difference seems to have been caused by chance and we statistically controlled for it. Possibly, the intervention influenced the intervention group and waitlist control group differentially. For example, mothers with higher level of depression and anxiety needed to focus more on the self and internalizing symptoms, while the waitlist control group may have had more mental space to focus on the parent-child relationship. Another possibility is that practicing formal meditations in between sessions was more feasible for mothers with lower levels of depression and anxiety, which gave them the opportunity to benefit more from the training.

A second limitation was the relatively low proportion of eligible women that chose to participate, in addition to the low adherence to the intervention. This seems to suggest that an investigation on the feasibility and acceptance of the current version of the online mindful parenting training is needed, as well as an adjustment of the current training in order to improve feasibility and acceptance for mothers with elevated levels of stress. Intention-to-treat analyses, however, showed that despite the low adherence, the training had some positive effects on the participants. This brings up the question how many sessions are needed to experience positive effects. The low percentage of eligible women that chose to participate, and thus the lower than intended sample size had negative consequences for the power of the current study. In combination with the non-clinical sample that the training was offered to, and the relatively small effects that were expected because the training was offered online and without professional or peer support, this may have limited the possibility of finding significant interaction effects of group and measurement occasion on some outcome measures. A third limitation is that the mindful parenting measure showed a weak internal consistency, resulting in being removed as an outcome measure. Therefore, it is not possible to confirm that the changes were due to an increase in mindful parenting. The low reliability of the measure may have been due to the fact that we chose the original (short) version of the IM-P, that also showed weak reliability in an earlier study on the effectiveness of Mindful Parenting (Potharst et al., in press a). A fourth limitation is the sole use of self-report measures. For a reliable measurement of parent-child interaction (that includes parental overreactivity), parent-child interaction observation is the preferred method (Miron et al., 2009).

The variability of significant results, the lack of information on the working mechanisms, and the relatively small effect size improvements that were shown in the current study call for modesty in the conclusions that are drawn. However, results do show that an online mindful parenting training seems to be effective in improving maternal symptoms of depression and anxiety, over-reactive parenting discipline, self-compassion, and mother-perceived child behavior. The current study does therefore provide first evidence that an online parenting training may be an easily accessible and valuable addition to the existing range of interventions for mothers with elevated levels of parental stress.

## Ethics Statement

This trial is registered in the Dutch Trial Register (NTR7401) and was approved of by the Ethics Committee of the University of Amsterdam. All participants provided written informed consent.

## Author Contributions

EP contributed to the development of the training, study design, data analysis, and wrote parts of the manuscript. MB contributed to data collection, data analysis, and wrote parts of the manuscript. IC, KvB, and AJ contributed to data collection. VS, IN, and SB contributed to the development of the training and to the study design. VP is the principal investigator and acquired funding, contributed to the study design, and monitored the process of data collection and analysis. All authors contributed to the final version of the manuscript.

## Conflict of Interest Statement

SB published books about mindful parenting, and EP published a book in Dutch about mindful parenting for parents with a baby. The remaining authors declare that the research was conducted in the absence of any commercial or financial relationships that could be construed as a potential conflict of interest.

## Footnotes

1 https://www.mindful-ouderschap.nl/

## References

[B1] Abidin R. R. (1983). *Parenting Stress Index Manual.* Charlottesville: Pediatric Psychology Press.

[B2] Achenbach T. Rescorla L. (2000). *Manual for the ASEBA Preschool Forms and Profiles.* Burlington: University of Vermont.

[B3] Aikens K. A. Astin J. Pelletier K. R. Levanovich K. Baase C. M. Park Y. Y. (2014). Mindfulness goes to work: impact of an online workplace intervention. *J. Occup. Environ. Med.* 56 721–731. 10.1097/JOM.0000000000000209 24988100

[B4] Anthony L. G. Anthony B. J. Glanville D. N. Naiman D. Q. Waanders C. Shaffer S. (2005). The relationships between parenting stress, parenting behaviour and preschoolers’ social competence and behaviour problems in the classroom. *Inf. Child Dev.* 14 133–154. 10.1002/icd.385

[B5] Arnold D. S. O’Leary S. G. Wolff L. S. Acker M. M. (1993). The Parenting Scale: A measure of dysfunctional parenting in discipline situations. *Psychol. Assess.* 5 137–144. 10.1037/1040-3590.5.2.137

[B6] Bagiella E. Sloan R. P. Heitjan D. F. (2000). Mixed-effects models in psychophysiology. *Psychophysiology* 37 13–20. 10.1111/1469-8986.3710013 10705763

[B7] Beer M. Ward L. Moar K. (2013). The relationship between mindful parenting and distress in parents of children with an autism spectrum disorder. *Mindfulness* 4 102–112. 10.1007/s12671-012-0192-4 24679352

[B8] Bernier A. Carlson S. M. Whipple N. (2010). From external regulation to self-regulation: early parenting precursors of young children’s executive functioning. *Child Dev.* 81 326–339. 10.1111/j.1467-8624.2009.01397.x 20331670

[B9] Bögels S. Restifo K. (2013). *Mindful Parenting: A Guide for Mental Health Practitioners.* New York, NY: Springer Science & Business Media.

[B10] Bögels S. M. Hellemans J. van Deursen S. Römer M. van der Meulen R. (2014). Mindful parenting in mental health care: effects on parental and child psychopathology, parental stress, parenting, coparenting, and marital functioning. *Mindfulness* 5 536–551. 10.1007/s12671-013-0209-7

[B11] Bornstein M. H. Hahn C. S. Haynes O. M. (2010). Social competence, externalizing, and internalizing behavioral adjustment from early childhood through early adolescence: developmental cascades. *Dev. Psychopathol.* 22 717–735. 10.1017/S0954579410000416 20883577 PMC3412561

[B12] Briggs-Gowan M. J. Carter A. S. Bosson-Heenan J. Guyer A. E. Horwitz S. M. (2006). Are infant-toddler social-emotional and behavioral problems transient? *J. Am. Acad. Child Adolesc. Psychiatry* 45 849–858. 10.1097/01.chi.0000220849.48650.59 16832322

[B13] Bugental D. B. Johnston C. (2000). Parental and child cognitions in the context of the family. *Annu. Rev. Psychol.* 51 315–344. 10.1146/annurev.psych.51.1.315 10751974

[B14] Cavanagh K. Strauss C. Cicconi F. Griffiths N. Wyper A. Jones F. (2013). A randomised controlled trial of a brief online mindfulness-based intervention. *Behav. Res. Ther.* 51 573–578. 10.1016/j.brat.2013.06.003 23872699

[B15] Ciciolla L. Gerstein E. D. Crnic K. A. (2014). Reciprocity among maternal distress, child behavior, and parenting: transactional processes and early childhood risk. *J. Clin. Child Adolesc. Psychol.* 43 751–764. 10.1080/15374416.2013.812038 23819445 PMC3808475

[B16] Cohen J. (1988). *Statistical Power Analysis for the Behavioral Sciences.* Hillsdale: Lawrence Erlbaum Associates.

[B17] Cole P. M. Martin S. E. Dennis T. A. (2004). Emotion regulation as a scientific construct: methodological challenges and directions for child development research. *Child Dev.* 75 317–333. 10.1111/j.1467-8624.2004.00673.x 15056186

[B18] Crnic K. A. Gaze C. Hoffman C. (2005). Cumulative parenting stress across the preschool period: relations to maternal parenting and child behaviour at age 5. *Inf. Child Dev.* 14 117–132. 10.1002/icd.384

[B19] Crugnola C. R. Ierardi E. Ferro V. Gallucci M. Parodi C. Astengo M. (2016). Mother-infant emotion regulation at three months: the role of maternal anxiety, depression, and parenting stress. *Psychopathology* 49 285–294. 10.1159/000446811 27410251

[B20] De Bruin E. I. Zijlstra B. J. Geurtzen N. van Zundert R. M. van de Weijer-Bergsma E. Hartman E. E. (2014). Mindful parenting assessed further: psychometric properties of the dutch version of the interpersonal mindfulness in parenting scale (IM-P). *Mindfulness* 5 200–212. 10.1007/s12671-012-0168-4 25126133 PMC4130420

[B21] Deater-Deckard K. (1998). Parenting stress and child adjustment: some old hypotheses and new questions. *Clin. Psycho. Sci. Prac.* 5 314–332. 10.1111/j.1468-2850.1998.tb00152.x

[B22] Deater-Deckard K. (2005). Parenting stress and children’s development: introduction to the special issue. *Inf. Child Dev.* 14 111–115. 10.1002/icd.383

[B23] Duncan L. G. (2007). *Assessment of Mindful Parenting Among Parents of Early Adolescents: Development and Validation of the Interpersonal Mindfulness in Parenting Scale.* State College, PA: The Pennsylvania State University. [Doctoral dissertation’s thesis].

[B24] Duncan L. G. Coatsworth J. D. Greenberg M. T. (2009). A model of mindful parenting: implications for parent–child relationships and prevention research. *Clin. Child Fam. Psychol. Rev.* 12 255–270. 10.1007/s10567-009-0046-3 19412664 PMC2730447

[B25] Early Child Care Research Network. [NICHD] (2004). Affect dysregulation in the mother–child relationship in the toddler years: antecedents and consequences. *Dev. Psychopathol.* 16 43–68. 15115064 10.1017/s0954579404044402

[B26] Emerson L. M. Aktar E. De Bruin E. Potharst E. Bögels S. (in press). Mindful parenting in secondary child mental health: key parenting predictors of treatment effects. *Mindfulness.* 10.1007/s12671-019-01176-w

[B27] Feldman R. Eidelman A. I. (2009). Biological and environmental initial conditions shape the trajectories of cognitive ansocial-emotiondevelopment across the first years of life. *Dev. Sci.* 12 194–200. 10.1111/j.1467-7687.2008.00761.x 19120428

[B28] George D. Mallery P. (2014). *IBM SPSS Statistics 21 step: A Simple Guide and Reference*, 13th Edn. Boston: Pearson.

[B29] Glück T. M. Maercker A. (2011). A randomized controlled pilot study of a brief web-based mindfulness training. *BMC Psychiatry* 11:175. 10.1186/1471-244X-11-175 22067058 PMC3250944

[B30] Gouveia M. J. Carona C. Canavarro M. C. Moreira H. (2016). Self-compassion and dispositional mindfulness are associated with parenting styles and parenting stress: The mediating role of mindful parenting. *Mindfulness* 7 700–712. 10.1007/s12671-016-0507-y

[B31] Horton T. V. Wallander J. L. (2001). Hope and social support as resilience factors against psychological distress of mothers who care for children with chronic physical conditions. *Rehabil. Psychol.* 46 382–399. 10.1037/0090-5550.46.4.382

[B32] Ingersoll B. Wainer A. L. Berger N. I. Pickard K. E. Bonter N. (2016). Comparison of a self-directed and therapist-assisted telehealth parent-mediated intervention for children with ASD: A pilot RCT. *J. Autism Dev. Disord.* 46 2275–2284. 10.1007/s10803-016-2755-z 26922192

[B33] Kabat-Zinn J. (1990). *Full Catastrophe Living: The Program of the Stress Reduction Clinic at the University of Massachusetts Medical Center.* New York, NY: Delta.

[B34] Kabat-Zinn M. Kabat-Zinn J. (1997). *Everyday Blessings: the Inner Work of Mindful Parenting.* New York, NY: Hyperion.

[B35] Kersten-Alvarez L. E. Hosman C. M. Riksen-Walraven J. M. Van Doesum K. Hoefnagels C. (2011). Which preventive interventions effectively enhance depressed mothers’ sensitivity? a meta-analysis. *Infant Ment. Health J.* 32 362–376. 10.1002/imhj.20301 28520142

[B36] Kroenke K. Spitzer R. L. Williams J. B. Monahan P. O. Löwe B. (2007). Anxiety disorders in primary care: prevalence, impairment, comorbidity, and detection. *Ann. Intern. Med.* 146 317–325. 10.7326/0003-4819-146-5-200703060-00004 17339617

[B37] Kroenke K. Spitzer R. L. Williams J. B. W. (2003). The patient health questionnaire- 2: validity of a two-item depression screener. *Med. Care* 41 1284–1292. 10.1097/01.MLR.0000093487.78664.3C 14583691

[B38] Kroenke K. Spitzer R. L. Williams J. B. W. Löwe B. (2009). An ultra-brief screening scale for anxiety and depression: the PHQ–4. *Psychosomatics* 50 613–621. 10.1176/appi.psy.50.6.613 19996233

[B39] Leerkes E. M. Su J. Calkins S. D. O’Brien M. Supple A. J. (2017). Maternal physiological dysregulation while parenting poses risk for infant attachment disorganization and behavior problems. *Dev. Psychopathol.* 29 245–257. 10.1017/S0954579416000122 26902983 PMC5654730

[B40] Lewallen A. C. Neece C. L. (2015). Improved social skills in children with developmental delays after parent participation in MBSR: the role of parent–child relational factors. *J. Child Fam. Stud.* 24 3117–3129. 10.1007/s10826-015-0116-8

[B41] Lipscomb S. T. Leve L. D. Shaw D. S. Neiderhiser J. M. Scaramella L. V. Ge X. (2012). Negative emotionality and externalizing problems in toddlerhood: overreactive parenting as a moderator of genetic influences. *Dev. Psychopathol.* 24 167–179. 10.1017/S0954579411000757 22293002 PMC3270900

[B42] Lorber M. F. (2012). The role of maternal emotion regulation in overreactive and lax discipline. *J. Fam. Psychol.* 26:642. 10.1037/a0029109 22888786 PMC4523636

[B43] Löwe B. Wahl I. Rose M. Spitzer C. Glaesmer H. Wingenfeld K. (2010). A 4-item measure of depression and anxiety: validation and standardization of the Patient Health Questionnaire-4 (PHQ-4) in the general population. *J. Affect. Disord.* 122 86–95. 10.1016/j.jad.2009.06.019 19616305

[B44] Lundahl B. Risser H. J. Lovejoy M. C. (2006). A meta-analysis of parent training: moderators and follow-up effects. *Clin. Psychol. Rev.* 26 86–104. 10.1016/j.cpr.2005.07.004 16280191

[B45] Mak W. W. Chan A. T. Cheung E. Y. Lin C. L. Ngai K. C. (2015). Enhancing Web-based mindfulness training for mental health promotion with the health action process approach: randomized controlled trial. *J. Med. Internet Res.* 17:e8. 10.2196/jmir.3746 25599904 PMC4319090

[B46] McMahon C. A. Meins E. (2012). Mind-mindedness, parenting stress, and emotional availability in mothers of preschoolers. *Early Childhood Res.* 27 245–252. 10.1016/j.ecresq.2011.08.002

[B47] Meppelink R. de Bruin E. I. Wanders-Mulder F. H. Vennik C. J. Bögels S. M. (2016). Mindful parenting training in child psychiatric settings: heightened parental mindfulness reduces parents’ and children’s psychopathology. *Mindfulness* 7 680–689. 10.1007/s12671-016-0504-1 27217845 PMC4859846

[B48] Milgrom J. Ericksen J. McCarthy R. Gemmill A. W. (2006). Stressful impact of depression on early mother–infant relations. *Stress Health* 22 229–238. 10.1002/smi.1101

[B49] Miller-Lewis L. R. Baghurst P. A. Sawyer M. G. Prior M. R. Clark J. J. Arney F. M. (2006). Early childhood externalising behaviour problems: Child, parenting, and family-related predictors over time. *J. Abnorm. Child Psychol.* 34 886–901. 10.1007/s10802-006-9071-6 17103309

[B50] Mills-Koonce W. R. Appleyard K. Barnett M. Deng M. Putallaz M. Cox M. (2011). Adult attachment style and stress as risk factors for early maternal sensitivity and negativity. *Infant Ment. Health J.* 32 277–285. 10.1002/imhj.20296 24855326 PMC4026358

[B51] Miron D. Lewis M. L. Zeanah C. H. (2009). “Clinical use of observational procedures in early childhood relationship assessment,” in *Handbook of Infant Mental Health*, ed. Zeanah C. H. (New York, NY: Guilford Press), 252–265.

[B52] Morgan J. K. Izard C. E. Hyde C. (2014). Emotional reactivity and regulation in Head Start children: links to ecologically valid behaviors and internalizing problems. *Soc. Dev.* 23 250–266. 10.1111/sode.12049 25067866 PMC4106433

[B53] Morledge T. J. Allexandre D. Fox E. Fu A. Z. Higashi M. K. Kruzikas D. T. (2013). Feasibility of an online mindfulness program for stress management—a randomized, controlled trial. *Ann. Behav. Med.* 46 137–148. 10.1007/s12160-013-9490-x 23632913 PMC3775158

[B54] Morris A. S. Silk J. S. Steinberg L. Myers S. S. Robinson L. R. (2007). The role of the family context in the development of emotion regulation. *Soc. Dev.* 16 361–388. 10.1111/j.1467-9507.2007.00389.x 19756175 PMC2743505

[B55] Murray L. Cooper P. Fearon P. (2014). Parenting difficulties and postnatal depression: implications for primary healthcare assessment and intervention. *Commu. Pract.* 87 34–38. 25612413

[B56] Neece C. L. Green S. A. Baker B. L. (2012). Parenting stress and child behavior problems: a transactional relationship across time. *Am. J. Intellect. Dev. Disabil.* 117 48–66. 10.1352/1944-7558-117.1.48 22264112 PMC4861150

[B57] Neff K. (2003). Self-compassion: an alternative conceptualization of a healthy attitude toward oneself. *Self Identity* 2 85–101. 10.1080/15298860390129863

[B58] Neff K. D. (2003). The development and validation of a scale to measure self-compassion. *Self Identity* 2 223–250. 10.1080/15298860309027 26979311

[B59] Neff K. D. Faso D. J. (2015). Self-compassion and well-being in parents of children with autism. *Mindfulness* 6 938–947. 10.1007/s12671-014-0359-2

[B60] Nieuwboer C. C. Fukkink R. G. Hermanns J. M. (2013). Online programs as tools to improve parenting: a meta-analytic review. *Child. Youth Serv. Rev.* 35 1823–1829. 10.1016/j.childyouth.2013.08.008

[B61] O’Leary S. G. Slep A. M. S. Reid M. J. (1999). A longitudinal study of mothers’ overreactive discipline and toddlers’ externalizing behavior. *J. Abnorm. Child Psychol.* 27 331–341. 10582835 10.1023/a:1021919716586

[B62] Östberg M. Hagekull B. (2000). A structural modeling approach to the understanding of parenting stress. *J. Clin. Child Adolesc. Psychol.* 29 615–625. 10.1207/S15374424JCCP2904_13 11126638

[B63] Östberg M. Hagekull B. Hagelin E. (2007). Stability and prediction of parenting stress. *Inf. Child Dev.* 16 207–223. 10.1002/icd.516 8163775

[B64] Potharst E. S. Baartmans J. M. Bögels S. M. (in press a). Mindful parenting training in a clinical versus non-clinical setting: an explorative study. *Mindfulness* 10.1007/s12671-018-1021-1

[B65] Potharst E. S. Zeegers M. Bögels S. M. (in press b). Mindful with your toddler group training: feasability, acceptibility, and effects on subjective and objective measures. *Mindfulness* 10.1007/s12671-018-1073-2

[B66] Prinzie P. Onghena P. Hellinckx W. (2007). Reexamining the Parenting Scale: Reliability, factor structure, and concurrent validity of a scale for assessing the discipline practices of mothers and fathers of elementary-school-aged children. *Eur. J. Psychol. Assess.* 23 24–31. 10.1027/1015-5759.23.1.24

[B67] Raes F. Pommier E. Neff K. D. Van Gucht D. (2011). Construction and factorial validation of a short form of the self-compassion scale. *Clin. Psychol. Psychother.* 18 250–255. 10.1002/cpp.702 21584907

[B68] Roemer L. Williston S. K. Rollins L. G. (2015). Mindfulness and emotion regulation. *Curr. Opin. Psychol.* 3 52–57. 10.1016/j.copsyc.2015.02.006

[B69] Rutherford H. J. Wallace N. S. Laurent H. K. Mayes L. C. (2015). Emotion regulation in parenthood. *Dev. Rev.* 36 1–14. 10.1016/j.dr.2014.12.008 26085709 PMC4465117

[B70] Siegel D. J. Hartzell M. (2003). *Parenting From the Inside Out: how Deeper Self-Understanding can Help you Raise Children who Thrive.* New York, NY: J.P. Tarcher/Putnam.

[B71] Singh N. N. Lancioni G. E. Winton A. S. Singh J. Curtis W. J. Wahler R. G. (2007). Mindful parenting decreases aggression and increases social behavior in children with developmental disabilities. *Behav. Modif.* 31 749–771. 10.1177/0145445507300924 17932234

[B72] Spijkerman M. P. J. Pots W. T. M. Bohlmeijer E. T. (2016). Effectiveness of online mindfulness-based interventions in improving mental health: a review and meta-analysis of randomised controlled trials. *Clin. Psychol. Rev.* 45 102–114. 10.1016/j.cpr.2016.03.009 27111302

[B73] Truijens S. E. M. Meems M. Kuppens S. M. I. Broeren M. A. C. Nabbe K. C. A. M. Wijnen H. A. (2014). The HAPPY study (holistic approach to pregnancy and the first postpartum year): design of a large prospective cohort study. *BMC Preg. Childbirth* 14:312. 10.1186/1471-2393-14-312 25201155 PMC4162933

[B74] Veerman J. W. Kroes G. De Meyer R. E. Nguyen L. M. Vermulst A. A. (2014). Opvoedingsbelasting in kaart gebracht. Een kennismaking met de opvoedingsbelastingvragenlijst (OBVL). *JGZ Tijdschrift voor Jeugdgezondheidszorg* 46 51–55. 10.1007/s12452-014-0016-0

[B75] Vermulst A. Kroes G. De Meyer R. Nguyen L. Veerman J. W. (2012). *Opvoedingsbelastingvragenlijst (OBVL). Handleiding.* Nijmegen: Praktikon.

[B76] Williams J. M. G. Crane C. Barnhofer T. Brennan K. Duggan D. S. Fennell M. J. (2014). Mindfulness-based cognitive therapy for preventing relapse in recurrent depression: a randomized dismantling trial. *J. Consult. Clin. Psychol.* 82 275–286. 10.1037/a0035036 24294837 PMC3964149

